# Quantifying and Benchmarking Disparities in COVID-19 Vaccination Rates by Race and Ethnicity

**DOI:** 10.1001/jamanetworkopen.2021.30343

**Published:** 2021-10-20

**Authors:** Marissa B. Reitsma, Jeremy D. Goldhaber-Fiebert, Joshua A. Salomon

**Affiliations:** 1 Department of Health Policy, Stanford University School of Medicine, Stanford, California

## Abstract

This decision analytical model quantifies disparities in uptake of COVID-19 vaccination by race and ethnicity and models alternative scenarios of persistent differential uptake and reduced barriers to access.

## Introduction

Unequal COVID-19 vaccination rates in the United States have compounded existing disparities in cases, hospitalizations, and deaths among Black and Hispanic populations.^1,2,3^ In this study, we quantify how differential vaccine uptake by race and ethnicity within each US state produced substantial vaccination coverage disparities during the initial scale-up among older adults. We model alternative scenarios for the period after eligibility opened to all adults, including a scenario of persistent differential uptake and scenarios that include efforts to reduce disparities by addressing access barriers, increasing vaccine confidence, and prioritizing disadvantaged geographic areas.

## Methods

For this decision analytical model, we analyzed demographic data (population distribution by age, self-reported race and ethnicity, and census tract) from the American Community Survey.^4^ From state websites, we extracted shares of people receiving at least 1 vaccine dose, stratified by age and separately by self-reported race and ethnicity, through March 31, 2021 (eMethods in the Supplement). We extracted and analyzed data for American Indian or Alaska Native, Asian, Black, Hispanic, Multiracial, Native Hawaiian or Other Pacific Islander, and White populations, but we only report estimates for Asian, Black, Hispanic, and White populations based on our minimum state-level population threshold of 200 000 people. Combining these data, we estimated relative uptake rates for the initial scale-up preceding all-adult eligibility by race and ethnicity within each state. We defined relative uptake rates as the observed share of vaccinations for a racial or ethnic group divided by the expected share if uptake across racial and ethnic groups within each age group were proportional to population size. This approach allowed us to control for the interaction of age-based eligibility criteria with differing age structures by race and ethnicity and thereby isolate the effects of differential vaccination accessibility and confidence.

We modeled vaccination scale-up within each census tract in a state under 3 scenarios. In the persistent differential uptake scenario, we used observed daily vaccination rates by state reported by the Centers for Disease Control and Prevention between April 1 and July 1, 2021, and assumed disparities in state-specific relative uptake rates by race and ethnicity would continue. In the equalized uptake scenario, we assumed equal vaccination rates in all racial and ethnic groups within a state, set each day as the highest rate observed for any group with a state-level population of at least 200 000 people. In the equalized uptake and geographic targeting scenario, we modeled the further impact of doubling vaccination rates in the most disadvantaged quartile of census tracts (according to the Centers for Disease Control and Prevention’s Social Vulnerability Index) during 6 weeks beginning April 1. We compared projections in the 3 scenarios to estimated levels of actual coverage by race and ethnicity as of July 1 to benchmark progress toward addressing vaccine equity.^5^

All analyses were conducted using R version 4.0.3 (R Project for Statistical Computing). We did not seek institutional review board approval because our analysis only included deidentified, publicly available data that are considered exempt under the Common Rule.

## Results

**Figure 1.**
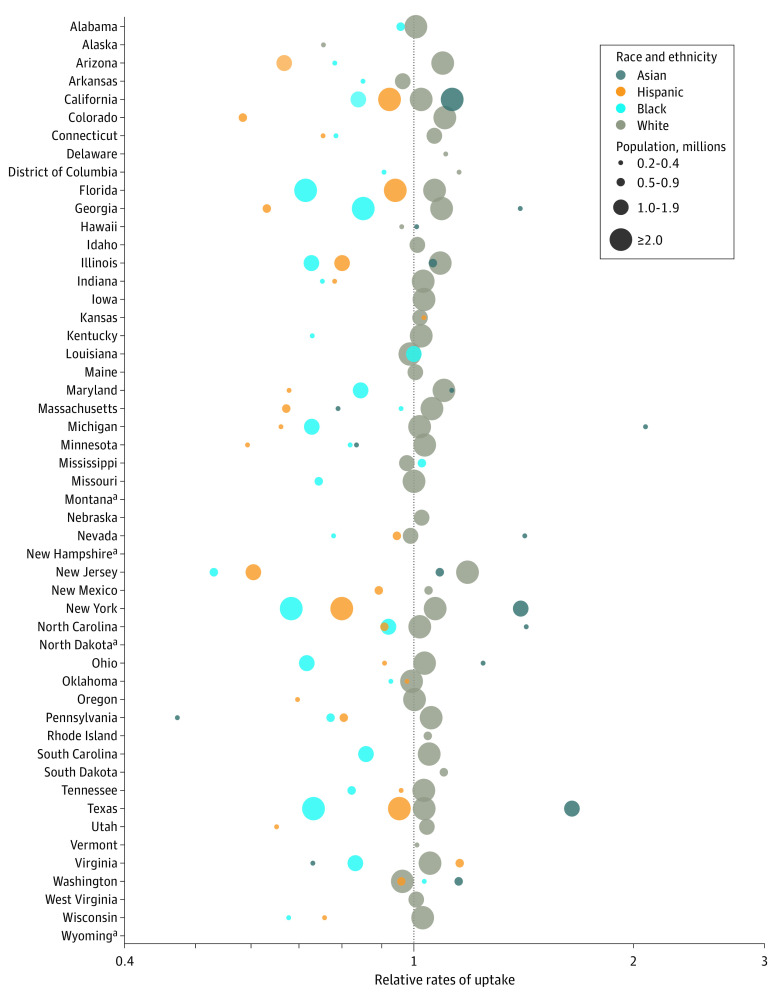
Relative Rates of COVID-19 Vaccination Uptake by Race and Ethnicity and State, Visualized on a Log Scale Estimates are shown for populations that exceed 200 000 people and have data available on state reporting dashboards. Relative rates of uptake are defined as the observed share of vaccinations for a racial or ethnic group, divided by the expected share if uptake across racial and ethnic groups within each age group were proportional to population size. ^a^State data not reported by race and ethnicity as of March 31, 2021.

In most states, relative uptake rates through March 31, 2021, were substantially higher among White compared with Black and Hispanic adults, by a median factor of 1.3 times for White compared with Black adults (IQR, 1.2-1.4 times) and a median 1.3 times for White compared with Hispanic adults (IQR, 1.1-1.6 times) (Figure 1). Combined effects of disproportionate uptake and age-based eligibility resulted in estimated coverage among Black and Hispanic adults (29%) being one-third lower than among White adults (43%) by the end of March.

**Figure 2.**
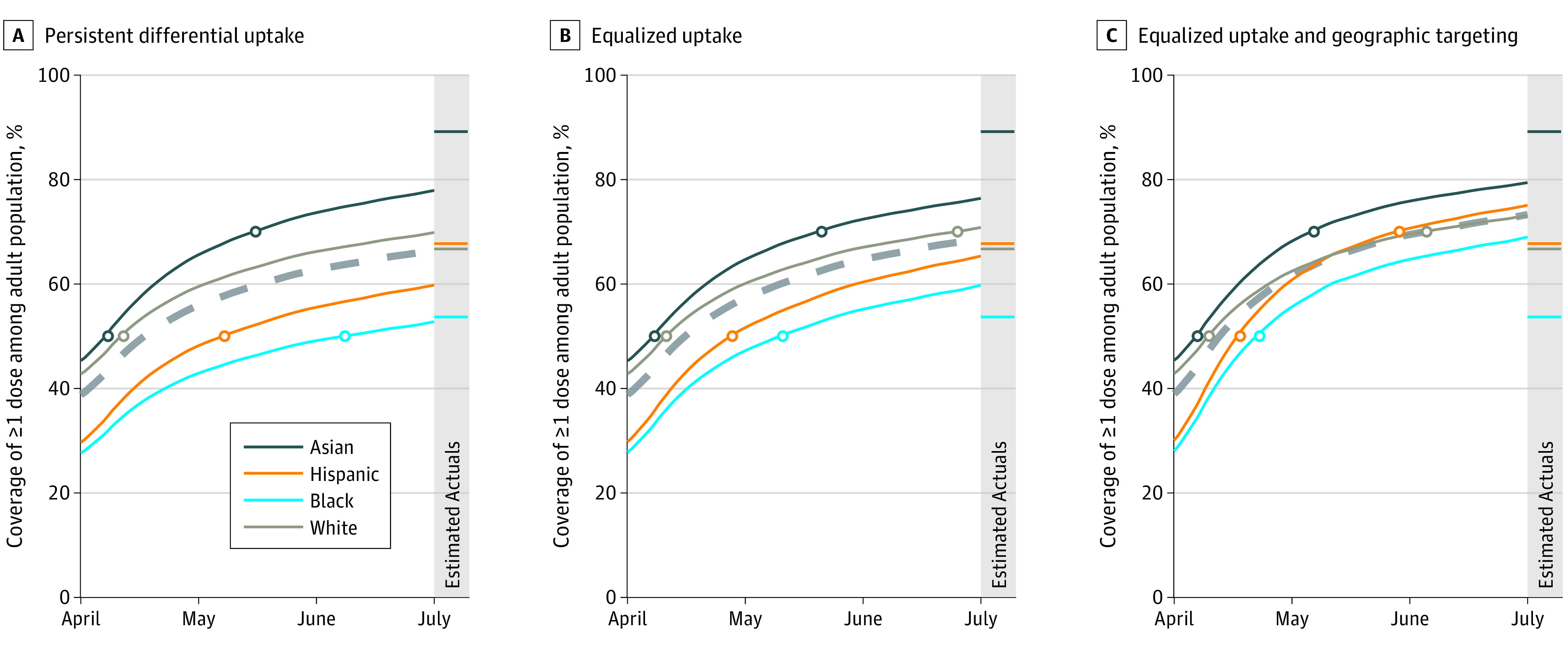
Coverage of 1 or More COVID-19 Vaccination Doses Among Population 18 Years and Older, by Racial or Ethnic Group, Aggregated to National Level Panels show persistent differential uptake (A), equalized uptake (B), and equalized uptake and geographic targeting (C). Dashed lines show overall coverage among the US population aged 18 years or older.

In the persistent differential uptake scenario, Hispanic and Black adults would reach 50% coverage of at least 1 vaccine dose nationally 57 days and 26 days later, respectively, than White adults. In the equalized uptake scenario, delays would be reduced to 30 days for Black adults and 17 days for Hispanic adults. In the equalized uptake and geographic targeting scenario, delays to 50% coverage would narrow to 13 and 8 days for Black and Hispanic adults, respectively; in this scenario, coverage disparities between Hispanic and White adults would have been eliminated by July 1, 2021, and the coverage gap would have been reduced by 76% for Black adults (Figure 2).

Actual levels of coverage nationally on July 1 were estimated to be 68%, 67%, and 54% for Hispanic, White, and Black adults, respectively (Figure 2). Actual coverage among Black adults reached levels projected in the equalized uptake scenario in only 10 of 30 states with reported data and sufficient population size, whereas coverage among Hispanic adults reached these benchmark levels in 20 of 27 states analyzed.

## Discussion

The disparities in vaccination among Black and Hispanic adults seen in this study highlight the urgent need to invest in policies and interventions to promote vaccine equity. Our results additionally demonstrate the benefits of place-based targeting of efforts to promote vaccination uptake. Limitations of this analysis include incomplete reporting of race and ethnicity in state vaccination data and lack of these data at the substate level. Nevertheless, by applying consistent rules, we reconciled heterogeneous reporting data to quantify vaccination disparities and demonstrated the need for equity-focused policies to ensure that underserved communities are not left behind.^6^

## Notes

Supplement. **eMethods.** Supplementary Methods
