# Error in Results

**DOI:** 10.1001/jamanetworkopen.2021.38575

**Published:** 2021-11-11

In the Research Letter titled “Quantifying and Benchmarking Disparities in COVID-19 Vaccination Rates by Race and Ethnicity,”^1^ published October 20, 2021, in the first sentence of the second paragraph in the Results, the phrase “Black and Hispanic adults” was incorrectly transposed as “Hispanic and Black adults.” This article has been corrected.^1^
