# Supplementary material for: Quantifying and Benchmarking Disparities in COVID-19 Vaccination Rates by Race and Ethnicity
Source: JAMA Netw Open. 2021 Oct 20;4(10):e2130343. doi: 10.1001/jamanetworkopen.2021.30343 (PMC8529412; doi:10.1001/jamanetworkopen.2021.30343)
Supplement: Supplement. — eMethods. Supplementary Methods [file jamanetwopen-e2130343-s001.pdf]

Supplemental Online Content

Reitsma MB, Goldhaber-Fiebert JD, Salomon JA. Quantifying and benchmarking disparities in COVID-19 vaccination rates by race and ethnicity. *JAMA Netw Open.* 2021;4(10):e2130343. doi:10.1001/jamanetworkopen.2021.30343

eMethods. Supplementary Methods

This supplemental material has been provided by the authors to give readers additional information about their work.

© 2021 Reitsma MB et al. *JAMA Network Open.*

eMethods – Supplementary Methods

- Vaccination data for Hispanic adults in Alabama were treated as missing because ethnicity is missing for more than half of data. Data for Georgia were from March 22, 2021, as data on race/ethnicity stopped being reported due to a technical issue. Data for Indiana and Maine did not include individuals receiving the Janssen vaccine because the reporting structure for these states did not permit combining individuals receiving first-dose of a two-dose vaccine and individuals receiving the single-dose vaccine. Data for North Dakota were not included because the reporting structure does not permit computation of vaccine distribution by race/ethnicity. Montana, New Hampshire, North Dakota, and Wyoming did not report vaccine distribution by race/ethnicity as of March 31, 2021.

- The age distribution of vaccinations was unavailable from Arkansas, Georgia, Hawaii, Montana, New Hampshire, New York, and Wyoming as of March 31, 2021. We used the national distribution of vaccinations across age groups for these states.

- When state-specific data to permit estimation of relative uptake rates by race/ethnicity were unavailable, we used relative uptake rates estimated at the census division level. Racial/ethnic groups that did not have state-specific data available together comprised 2.0% of the United States population aged 18 years and older.

- We only report results for racial/ethnic groups with state-reported data and a minimum population size of 200,000 individuals.

- In cases where vaccination coverage data reported by CDC suggests a decrease in cumulative coverage (eg. due to reconciling reporting issues or changes in reporting definitions), we adjusted the daily vaccination data to enforce monotonically increasing cumulative coverage. For any decline in cumulative coverage, we replaced values from earlier dates that exceeded a lower coverage value from a later date with the lower coverage value. This allowed us to preserve the reported coverage levels as much as possible, while also ensuring that coverage increased monotonically. This adjustment impacted 76 out of 11,118 (0.6%) state-date combinations.

- We analyzed vaccination rates smoothed over a seven-day period to minimize the impact of day-of-week variations.

© 2021 Reitsma MB et al. *JAMA Network Open.*
